# Perioperative Management of a Patient With Multiple Comorbidities Undergoing Lung Resection for Bronchopulmonary Carcinoid Complicated by SARS-CoV-2 Infection: A Case Report

**DOI:** 10.7759/cureus.58310

**Published:** 2024-04-15

**Authors:** Despoina G Sarridou, Maria Konstantinidou, Afroditi Boutou, Giakoumis A Mitos, Theodoros Karaiskos

**Affiliations:** 1 Department of Anesthesiology and Intensive Care, Faculty of Medicine, School of Health Sciences, American Hellenic Educational Progressive Association University Hospital, Aristotle University of Thessaloniki, Thessaloniki, GRC; 2 Department of Respiratory Medicine, "George Papanikolaou" General Hospital, Thessaloniki, GRC; 3 Department of Respiratory Medicine, "Hippokration" General Hospital, Thessaloniki, GRC; 4 Department of Anesthesiology and Intensive Care, American Hellenic Educational Progressive Association University Hospital, Aristotle University of Thessaloniki, Thessaloniki, GRC; 5 Department of Cardiothoracic Surgery, "George Papanikolaou" General Hospital, Thessaloniki, GRC

**Keywords:** hospital-acquired covid-19 infection, thoracotomy, multiple myeloma, sars-cov-2 (severe acute respiratory syndrome coronavirus -2), pulmonary carcinoid tumor

## Abstract

We report a case of a high-risk patient with multiple comorbidities who underwent right median lobectomy and lymph node resection due to a carcinoid tumor. The patient’s course was complicated by a hospital-acquired severe acute respiratory syndrome coronavirus 2 (SARS-CoV-2) infection and a postoperative chest hematoma requiring urgent thoracotomy. Multidisciplinary and timely management resulted in a favorable patient outcome.

## Introduction

Bronchopulmonary carcinoids are rare and slow-growing neuroendocrine malignancies, accounting for less than 2% of lung tumors. The percentage of bronchopulmonary carcinoids that arise within the segmental or sub-segmental bronchi is 80-90%. Diagnosis relies on a contrast-enhanced chest computed tomography (CT) scan. Early diagnosis is extremely important as carcinoid tumors have poor response to radiotherapy and adjuvant chemo, making the complete resection of the tumor and the regional lymph nodes the main curative approach. Lung-saving surgeries are preferable, especially for tumors located peripherally. The outcome of typical carcinoid tumor surgery is excellent even for patients with local nodal metastasis, which does not preclude definitive surgical treatment. However, larger tumors require cautious follow-up postoperatively, as the chance of recurrence is relatively higher [1, 2].

The novel coronavirus disease 2019 (COVID-19) is a highly contagious viral infection causing severe acute respiratory coronavirus-2 syndrome (SARS-CoV-2). Its rapid spread and severe clinical presentation influence patient management in all specialties, including thoracic surgery. The increasing incidence of COVID-19, influences morbidity and mortality negatively, by exhibiting both organ-specific and systemic effects. In high-risk individuals admitted with chronic cardiorespiratory conditions and will be submitted to urgent surgery for thoracic malignancies, SARS-CoV-2 infections may increase the rate of postoperative complications, the length of stay in the ICU, morbidity, and mortality [3-5].

## Case presentation

**Figure 1:**
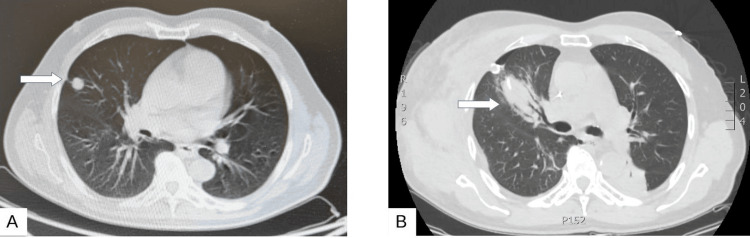
Preoperative CT scan. A) The atypical carcinoid is presented as a nodule in the lateral segment of the middle lobe (arrow). B) Postoperative CT scan. A chest tube and a small apex pneumothorax can be seen, along with lung consolidation of the right upper lobe (arrow) and a chest wall hematoma.

**Figure 2:**
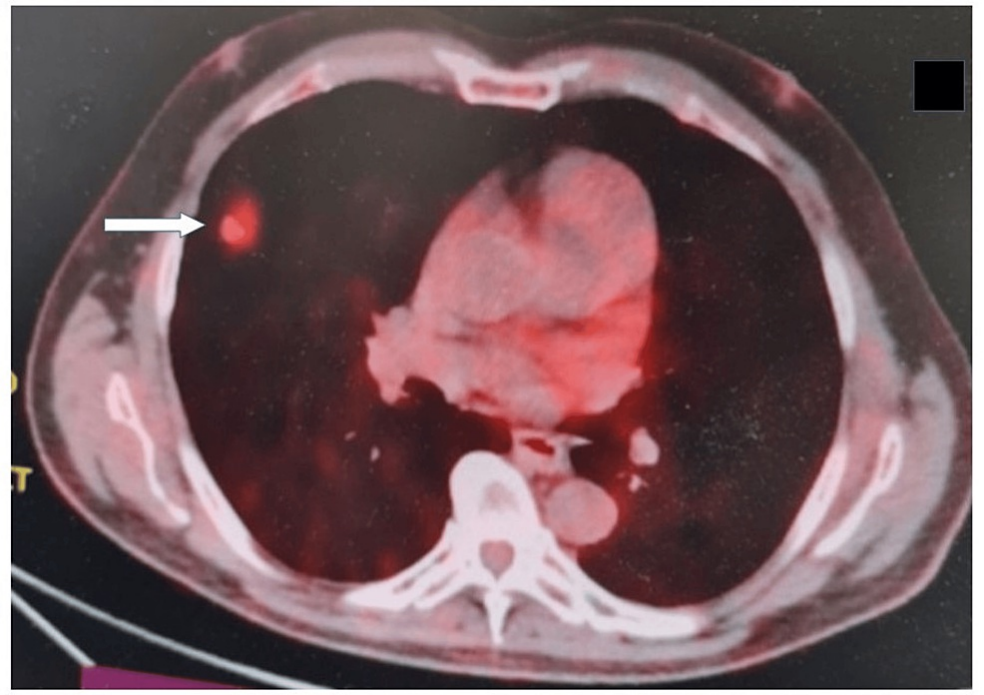
Preoperative PET/CT scan. The standardized uptake value of the atypical carcinoid (arrow) was 4.9.

A 59-year-old male American Society of Anesthesiology Physical Status (ASA-PS) III patient was diagnosed with an atypical carcinoid tumor of the right median lobe (Figure 1A). Diagnosis was suspected on the basis of conventional chest CT scan (Figure 1), followed by positron emitted tomography (PET/CT) scan (Figure 2), and was further confirmed with lung biopsies. Staging was characterized as T1N0. The patient was scheduled for right median lobectomy and lymph node resection, via open thoracotomy. Other comorbidities included multiple myeloma treated with chemotherapy 30 years ago, hypertension, chronic renal disease, and chronic anemia. His home-based drug therapy included allopurinol (200 mg od), ivabradine (2.5 mg bd), lacidipine (2 mg od), and tinzaparin (5000 IU) for deep vein thrombosis prophylaxis.

Perioperative anesthesia management

All the imaging results, referral letters, and biochemistry, clotting and full blood count results were reviewed at the preoperative assessment. The reverse transcriptase polymerase chain reaction (RT-PCR) test for SARS-CoV-2 was negative, 48 hours prior to surgery. There was a multidisciplinary meeting between clinicians prior to surgery and a postoperative analgesia plan was tailored as per the patient’s needs, based on the trust analgesia protocols. The patient underwent an open right median lobectomy under general anesthesia. Intubation and one-lung ventilation were uncomplicated. A left-sided double-lumen tube 41 Fr was used and correct positioning was confirmed by flexible bronchoscopy. Propofol and rocuronium were given for induction and anesthesia was maintained with sevoflurane. Airway pressures on one lung ranged between 27 and 35 cmH_2_O throughout the surgery. Our opioid choice was fentanyl due to the preexisting renal impairment. An intercostal block with 20 mL of ropivacaine 0.75% was given by the surgeons under direct vision and fentanyl patient-controlled analgesia (PCA) was provided for postoperative pain control. The neuromuscular blockade was reversed with sugammadex 400 mg and he was transferred uneventfully to the high-dependency unit for one-to-one care during the first 24 hours, as per protocol.

Perioperative events

Procedure was complicated by a chest wall hematoma 11.7 cm of diameter, confirmed with a new chest CT scan and showed signs of progression. The chest CT scan also identified the presence of a lung consolidation with air bronchogram on the left upper lobe, atelectatic areas on the right lower lobe, right upper lobe, and left upper lobe, the presence of right pneumothorax with chest drain in situ, and ground glass opacities on the apical part of right upper lobe (Figure 1B). Therefore, a new expedited thoracotomy was planned on day three postoperatively. Surprisingly, the patient was in good general condition based on the circumstances. The same anesthesia and induction plan was performed without the application of the intercostal block to reduce the risk of a new hematoma.

The patient underwent a second RT-PCR test for SASR-CoV-2 with an oro-nasopharyngeal swab, on day four after the first procedure which came back as positive. It is important to note that he had no symptoms of pneumonia before surgery. He was then transferred to the high-dependency respiratory unit (HDRU) of our hospital in generally good condition, initially asymptomatic and apyrexial. He presented with mild symptoms, such as low fever and mildly impaired gas exchange, within 48 hours. Arterial oxygen saturation was 90-92% at room air. Supplemental oxygen therapy with 3 L/min was given via nasal cannula and paracetamol and tramadol were added for pain relief along with beta-2 agonists (via nebulizers), acetylcysteine, and enoxaparin for deep vein thrombosis prophylaxis. On the third day of hospitalization in the HDRU, the chest drain was removed after thoracic surgical review. Gas exchange progressively normalized and there was a significant clinical improvement. The patient was discharged after seven days from the HDRU and a week later was uneventfully sent home. Two months after discharge, the patient was totally asymptomatic and in excellent clinical condition. The timeline of events is also presented in Table 1.

**Table 1:** Timeline of events (D: day). HDRU: high-dependency respiratory unit; PCR: polymerase chain reaction

D1 post-operation	Day of first procedure - HDRU admission
D3	Repeat CT scan - second thoracotomy
D5	PCR positive
D6	Transfer to acute respiratory failure unit
D10	Removal of right chest drain
D16	Discharge

## Discussion

Unfortunately, in our region, an increased incidence of in-hospital acquired SARS-CoV-2 infection was reported, during the peak of the second wave of the pandemic, in 2020. As far as the potential transmission route is regarded, it is rendered too difficult to depict the exact date of SARS-CoV-2 infection for this case. Regardless the strict visiting protocols, the patient contracted the virus either before admission or after maintaining, however, a negative preoperative RT-PCR test due to low viral load or during the recovery period in between his hospital stay [5]. The asymptomatic window between the contact and presentation of symptoms is up to 11 days. The diagnosis of COVID-19 infection was not suspected on the basis of the patient’s good clinical condition, which was minimally symptomatic, but was mainly based on chest CT imaging and was confirmed with RT-PCR testing. Chest CT has been identified as an efficient clinical diagnostic tool, as it is considered the first-line imaging modality for people with suspected COVID-19 who remain asymptomatic. It allows clinical practitioners to identify people for whom COVID-19 is highly suspected, even though their PCR results are negative [6]. CT images of asymptomatic cases with COVID-19 pneumonia have a variety of characteristic patterns. The predominant features are ground glass opacity (GGO) with peripheral distribution, unilateral location and, mostly involving one or two lobes, mixed GGO and consolidation, centrilobular nodules, architectural distortion, cavitation, tree-in-bud, bronchial wall thickening, reticulation, subpleural bands, traction bronchiectasis, intrathoracic lymph node enlargement, vascular enlargement in the lesion, and pleural effusions, often combined with subpleural curvilinear line, fine reticulation, air bronchogram, halo sign or vascular enlargement signs [7]. In particular, our patient's CT scan showed GGO, consolidation with air bronchogram, pleural effusions, as well as banded atelectasis. To avoid further in-hospital transmission, as soon as the patient was found to be SARS-CoV-2 positive, he was transferred to an isolated, single-bed room and all health professionals who were treating him used special personal protective equipment (PPE).

It also has to be noted that the SARS-CoV-2 variants that caused the second pandemic wave resulted in a wide spectrum of clinical manifestations, with a large absolute number of patients who were not vaccinated experiencing severe pneumonia and rapid progression to acute respiratory distress syndrome and/or multiple organ failure. In these patients, the equilibrium of the inflammatory response is a major determinant of survival. The impact of anesthesia on immune system modulation is complex. It may vary and include both pro-inflammatory and anti-inflammatory effects [8]. General anesthesia and other relevant factors, including the use of preoperative narcotic drugs, precipitates for reduced response to stress, with well-recognized immunosuppressing effects that may last up to six weeks after any surgical procedure, even in healthy individuals [9]. In our case, this is also exacerbated by the preexisting comorbidities, that is the history of hematology disease, hypertension, renal disease, and anemia.

Our patient had an increased risk of perioperative death due to a high ASA-PS score. The ASA-PS scoring system was developed by anesthesiologists for preoperative risk assessments, in order to predict perioperative adverse events, including mortality. In general, this score was not designed for use as a predictor of survival beyond the perioperative period, while most surgical series have shown a correlation between a high ASA-PS score and 7-, 14-, and 30-day mortality. Intriguingly, it has been reported that ASA-PS III patients show lower survival than those with ASA-PS I or II [10]. Moreover, the risk of perioperative death increased further for this patient, due to SARS-Cov-2 infection. Among seropositive patients surgically treated for SARS-CoV-2 lung complications, high-risk score [3] and thoracotomy [11] were associated with higher mortality in previous reports, and these conditions were both present in our patient. Furthermore, Gonfiotti et al. described a postoperative morbidity of 60% and a mortality of 40%, among five patients who underwent anatomic lung resection for cancer, while being SARS-CoV-2 positive [12].

In addition, more complications could have occurred due to the two procedures that occurred; cases that uneventfully underwent two thoracotomies are not commonly found in the literature. Open thoracotomy is a procedure that is frequently complicated with severe pain, atelectasis, and delayed recovery. Microbial pneumonia is not uncommon and in our case the recovery was complicated with the SARS-CoV-2 infection. Besides, the endotracheal intubation itself does not guarantee a complication-free procedure [3]. Even though the anesthetic management was quite effective with a combination of multimodal analgesia and regional techniques, the presence of hematoma limited our available options for the second procedure. Furthermore, other limitations due to chronic renal disease made the use of non-steroid anti-inflammatory drugs (even the selective COX-2 inhibitors) most unattracted.

## Conclusions

In this manuscript, we presented the case of an immunocompromised patient with lung carcinoid, who underwent two thoracotomies, while being infected with SARS-CoV-2, with favorable outcome. Our patient had a series of factors potentially contributing to bad outcome and high risk for mortality. General anesthesia also precipitates reduced stress response and is a strong factor for respiratory complications such as chest infection and pneumonia. Regardless of the increased morbidity risk, the patient defied all odds by recovering and being discharged home. High clinical suspicion and multidisciplinary patient management favored recovery and good outcomes.
